# Breast Lesion Detection for Ultrasound Images Using MaskFormer

**DOI:** 10.3390/s24216890

**Published:** 2024-10-27

**Authors:** Aashna Anand, Seungho Jung, Sukhan Lee

**Affiliations:** 1 High School, Korea International School, Seongnam 13543, Republic of Korea; aanand25@student.kis.or.kr; 2 Department of Electrical and Computer Engineering, Sungkyunkwan University, Suwon 16419, Republic of Korea; richardjung@skku.edu; 3 Department of Artificial Intelligence, Sungkyunkwan University, Suwon 16419, Republic of Korea

**Keywords:** breast lesion, ultrasound, benign, malignant, MaskFormer, deep learning

## Abstract

This study evaluates the performance of the MaskFormer model for segmenting and classifying breast lesions using ultrasound images, addressing ultrasound’s limitations. Ultrasound used for breast cancer detection faces challenges like low image contrast and difficulty in the detection of small or multiple lesions, further complicated by variability based on operator skill. Initial experiments with U-Net and other CNN-based models revealed constraints, such as early plateauing in model loss, which indicated suboptimal learning and performance. In contrast, MaskFormer demonstrated continuous improvement, achieving higher precision in breast lesion segmentation and significantly reducing both false positives and false negatives. Comparative analysis showed MaskFormer’s superior performance, with the highest precision and recall rates for malignant lesions and an overall mean average precision (mAP) of 0.943. The model’s ability to detect a diverse range of breast lesions, including those potentially missed by the human eye, especially by less experienced practitioners, underscores its potential. These findings suggest that integrating AI models like MaskFormer could greatly enhance ultrasound performance for breast cancer detection, providing reliable, operator-independent image analysis and potentially improving patient outcomes on a global scale.

## 1. Introduction

As per the World Health Organization (WHO), an estimated 2.3 million women were diagnosed with breast cancer in 2022 [1]. This staggering number underscores the widespread prevalence and significant impact of the disease on women worldwide. Additionally, breast cancer claimed the lives of approximately 670,000 women globally last year, highlighting the severity of the disease [2]. Whether in high-income or low-income regions, breast cancer remains a universal health challenge that requires our collective attention and action [3]. Therefore, the early detection and diagnosis of breast cancer are critical, as they facilitate prompt treatment initiation, which can significantly enhance survival rates by preventing the progression of the disease.

According to the American College of Radiology (ACR), the early detection of breast lesions—abnormalities in the breast tissue often presenting as lumps or swellings—can be achieved through various imaging modalities: mammography, magnetic resonance imaging (MRI), and ultrasound [4]. Each of these imaging modalities offers its own advantages and disadvantages.

Table 1 highlights The Breast Imaging Reporting and Data System (BI-RADS), a set of guidelines to standardize breast imaging reporting and assist radiologists in categorizing their findings [5]. Category 0 indicates that the mammography, ultrasound, or MRI is incomplete. Category 1 and 2 indicate normal and benign cases, respectively, and hence, they do not require any further evaluation. Category 3 indicates probably benign findings, with a low probability of being cancerous (less than 2%), but follow-up imaging is recommended. Category 4 indicates suspicious findings, with a probability of malignancy ranging from 2% to 95%, necessitating a biopsy for definitive confirmation. Category 5 signifies highly suggestive malignancy, with a probability greater than 95%, requiring an immediate biopsy. Category 6 indicates known, biopsy-proven malignancy, for which cancer has been confirmed by biopsy, and appropriate action is required. In summary, based on whether a biopsy is needed or not, it is crucial to determine whether the patient has normal (no actions required), benign (yearly follow-up), or suspicious/confirmed malignant (biopsy needed) findings.

The first imaging modality, mammography, is widely used and highly effective for the early detection of breast cancer, especially in women over 50 [6]. It can detect tumors that are too small to be felt. Despite this, mammography involves exposure to X-ray radiation. Moreover, it can be less effective in women with dense breast tissue and may sometimes result in false positives or false negatives [7]. False positives occur when a mammogram indicates the presence of cancer when there is none, leading to further biopsies [8,9]. On the other hand, false negatives happen when a mammogram fails to detect an existing cancer, consequently leading to misdiagnosis, potentially delaying the treatment required, and adversely affecting outcomes [10]. Additionally, the mammography procedure is painful for all women, which might discourage regular screenings.

Next, MRI is highly sensitive and capable of producing detailed images, making it particularly valuable for patients at high risk and for evaluating the extent of cancer [11]. However, MRI is associated with higher costs and often requires significant wait times before an appointment can be scheduled. Additionally, it may generate false positives, leading to unnecessary biopsies.

Lastly, ultrasound is a non-radiation imaging technique that is widely accessible and effective for distinguishing between solid and fluid-filled cysts [12]. Solid masses are dense tissue growths in the breast that can be benign (non-cancerous) or malignant (cancerous), while fluid-filled cysts are sac-like structures filled with fluid within the breast tissue, and they generally do not entail the risk of cancer [13]. Despite its numerous benefits, the main disadvantage of ultrasound is that its effectiveness can be influenced by the operator’s skill, introducing variability in diagnostic accuracy [14]. Specifically, less experienced practitioners may miss lesions, particularly when multiple lesions are present.

Incorporating artificial intelligence (AI) into ultrasound imaging can help address these limitations by reducing dependence on the operator’s skill and improving the detection and classification of lesions as normal, benign, or malignant [15]. AI-enhanced ultrasound systems can analyze images more consistently and accurately, providing valuable support to physicians, particularly those who are new or less experienced.

The following are leading companies providing ultrasound solutions that incorporate specific AI features for breast lesion detection. Table 2 ranks these solutions by their reported accuracy in sensitivity and specificity.

GE Healthcare’s Invenia Automated Breast Ultrasound System (ABUS) exhibits a sensitivity of approximately 93% and a specificity close to 80% [16]. This solution employs 3D ultrasound imaging to complement traditional mammography. Siemens Healthineers’ AI-Rad Companion Breast achieves a sensitivity of around 87% and a specificity of roughly 83% [17]. It offers automated analysis and integrates efficiently with existing imaging workflows. Philips’ AI Breast Ultrasound Solution demonstrates a sensitivity of approximately 88% and a specificity of about 83% [18].

Hologic’s Brevera Breast Biopsy System provides a sensitivity of about 85% and a specificity of around 80% [19]. This system combines real-time imaging and biopsy capabilities with AI algorithms for accurate lesion classification. Overall, all of these commercial models use AI, specifically including convolutional neural networks (CNNs), to enhance detection accuracy.

## 2. Related Works

In recent years, various studies have built upon the critical need for early breast cancer detection and classification through the integration of deep learning.

### 2.1. AI Solutions for Breast Lesion Detection

This study, based on CNN and ABUS, utilized a multicenter dataset to enhance the model’s robustness, achieving an internal accuracy of 78% and an overall accuracy of 71% when including data from all test centers [20]. However, its performance diminished for lesions smaller than 10 mm, underscoring the need for continued refinement in detecting small lesions.

Another study employed CNNs with patch-based U-Net and transfer learning across two datasets for breast lesion detection [21]. It achieved true positive fractions of 0.99 for dataset A and 0.92 for dataset B; the study highlighted the reduction in false negatives, which is crucial for accurate diagnosis.

In a different approach, researchers evaluated various deep learning architectures, including Fast R-CNN and Single Shot MultiBox Detector (SSD300), using a newly annotated dataset of 579 benign and 464 malignant cases [22]. Specifically, the study found that SSD300 achieved the best performance in terms of the average precision rate (APR) for lesion detection, while DenseNet proved the most effective in classification tasks, achieving a high APR.

Research incorporating the Viola Jones and YOLOv3 algorithms for breast lesion detection achieved diagnostic accuracy by enhancing the sensitivity and specificity of the detection process, reaching as high as 70% [23].

Another noteworthy study investigated the use of an anchor-free network for breast lesion detection from ultrasound images, enhanced through segmentation-based techniques. This method focused on the contrast enhancement of ultrasound images, followed by lesion classification using an anchor-free network, achieving a mean average precision (mAP) of 0.802 for the BUSI dataset [24]. The detection precision for benign and malignant lesions was 0.816 and 0.789, respectively, with recall values of 0.932 and 0.889.

In another novel approach, YOLOv3 was combined with enhanced feature extraction techniques for breast ultrasound tumor detection and classification. This method utilized an improved YOLOv3 algorithm integrating the ResNet and DenseNet architectures with the Darknet-53 framework, aiming to address the challenges of low contrast, blurred boundaries, and artifacts in ultrasound images [25]. Evaluated on a dataset of 3259 images, the model achieved a mAP of 0.745, with a benign precision of 0.851 and a malignant precision of 0.637.

### 2.2. Problem Statements

Despite significant advancements in breast cancer detection, accurately segmenting and classifying breast lesions using ultrasound imaging remains challenging due to the variability in lesion appearance and the inherent limitations of ultrasound, including operator dependency and difficulty in detecting complex lesions due to low image contrast. Existing AI-enhanced imaging solutions, while promising, often fall short in terms of consistency and accuracy, particularly for these challenging lesion types. Therefore, there is a pressing need to develop more reliable and precise AI-based models that can enhance the early detection and accurate diagnosis of breast cancer by aiding in the segmentation and classification of breast lesions in ultrasound images.

### 2.3. Contribution

In our proposed research, we aim to contribute to the field of breast lesion segmentation and classification for ultrasound images by harnessing the latest advancements in AI technology. Specifically, we focused on developing a MaskFormer-based segmentation and classification model. More precisely, our contributions are summarized below.

- We developed and implemented an innovative MaskFormer-based approach for segmenting and classifying breast lesions in ultrasound images. This method leverages state-of-the-art techniques in semantic segmentation to identify and classify breast lesions more accurately than traditional methods.
- We applied our MaskFormer-based model to the Breast Ultrasound Images (BUSI) dataset in order to evaluate its efficacy. The BUSI dataset, which includes various annotated breast ultrasound images, serves as a robust benchmark for assessing the performance of our model.
- Our research demonstrates significant improvements in diagnostic metrics, including sensitivity, specificity, and mean average precision (mAP). By systematically comparing our results with existing methods, we show that the MaskFormer model can reduce false negatives and positives, thereby enhancing diagnostic reliability. Our comprehensive evaluation reveals the model’s superior performance in malignant precision and recall rates, with a high mAP. The model’s robustness is further highlighted because of its ability to detect a variety of lesions that might be missed by the human eye, particularly by less experienced clinicians.

## 3. Materials and Methods

Our proposed research focuses on the integration of AI for breast lesion segmentation and classification using advanced image segmentation techniques and models. The methodology includes detailed explanations of the dataset, model development, evaluation metrics, software environment, and overall mechanism/architecture. Each of these aspects is elaborated on in subsequent sections.

### 3.1. Dataset

The dataset utilized in this study is derived from the Breast Ultrasound Image Dataset (BUSI) [26]. Originally collected from Baheya Hospital for Early Detection and Treatment of Women’s Cancer in Cairo, Egypt, BUSI comprises 891 benign images, 421 malignant images, and 266 normal images. To maintain consistency, the dataset was divided into training (80%), testing (10%), and validation (10%) sets using an 8:1:1 ratio, respectively.

### 3.2. Image Segmentation Techniques

Image segmentation is a crucial task in computer vision, especially in medical imaging, as it involves partitioning an image into meaningful segments in order to facilitate analysis and interpretation. This process can be broadly categorized into three types: instance segmentation, semantic segmentation, and panoptic segmentation.

Instance segmentation identifies and delineates each object instance in an image, which is essential for applications requiring distinguishing between individual objects of the same category. Semantic segmentation assigns a label to every pixel in an image based on its class, treating all objects of a class as a single entity. Meanwhile, panoptic segmentation combines both instance and semantic segmentation by labeling each pixel with a class and distinguishing between different instances of the same class [27].

Among these, semantic segmentation is most suitable for our research due to its ability to provide a detailed understanding of the entire scene context. This approach is particularly beneficial for distinguishing between areas where lesions are present and where they are not in ultrasound images. The accurate delineation of lesion boundaries is crucial for proper diagnosis, and by focusing on pixel-level segmentation, semantic segmentation ensures the comprehensive and precise analysis necessary for effectively separating the lesion from the rest of the image.

Several models have been developed for image segmentation, each with its own strengths and limitations. Some notable models include YOLOv3 (You Only Look Once, version 3), U-Net, and DeepLab.

YOLOv3 is designed primarily for object detection, and it performs segmentation by dividing images into a grid and predicting bounding boxes and class probabilities for each cell [28]. While YOLOv3 is fast and efficient, it is less suited to the pixel-level accuracy required in semantic segmentation. U-Net, a CNN widely used in biomedical image segmentation, excels in scenarios requiring detailed and precise segmentation but may struggle with more complex, real-world scenes [29]. DeepLab, developed by Google, employs dilated convolution to capture multi-scale context, offering high accuracy but at the cost of increased computational resources [30].

MaskFormer represents a significant advancement in computer vision, particularly for its innovative mask-classification framework [31]. This framework unifies both semantic and instance-level segmentation tasks, moving beyond traditional per-pixel classification methods. Instead of classifying individual pixels, MaskFormer predicts a set of binary masks, each associated with a global class label. This allows the model to capture contextual information and long-range dependencies across the image. These features make it highly effective for segmenting breast lesions in ultrasound images, where boundaries may be unclear due to low contrast or noise in the image; hence, we chose MaskFormer for segmentation in our research. MaskFormer’s transformer decoder can produce per-segment embeddings, in which each embedding corresponds to a distinct region or object within the image, which are combined with per-pixel features [31]. This enables the model to directly predict binary masks representing regions of interest without relying on pixel-by-pixel classification. This process can allow for a faster and more accurate detection of both small and large breast lesions in our research.

Furthermore, we researched MaskFormer’s application in ultrasound or medical imaging. We found an interesting article on the use of different transformer-based models including MaskFormer for the detection of different kinds of cancer cells in histopathological breast images. Research suggested that augmenting MaskFormer’s transformer-based architectures with advanced techniques like auto-augmentation can further enhance performance, particularly in medical imaging tasks [32]. This research showed that the MaskFormer-based model could reliably detect unclear cancer cells in histology slides, which might be missed during microscopic examination. Overall, MaskFormer’s advanced architecture and flexibility make it an ideal choice for our research on detecting subtle and unclear lesions in ultrasound images that might be missed by less experienced practitioners in ultrasound-based breast lesion detection.

### 3.3. Image Classification Techniques

The accurate classification of breast lesions is essential for determining appropriate patient treatment. Our study focused on classifying lesions into three categories: normal, benign, and malignant, based on the BI-RADS guidelines. Category 1 indicates normal findings, while categories 2 and 3 indicate benign findings, which typically do not require immediate action beyond regular monitoring. In contrast, Categories 4, 5, and 6 indicate suspicious or confirmed malignant lesions, necessitating a biopsy or further medical intervention.

Several models have been developed for image classification, each with its own advantages. Traditional models like support vector machines (SVMs) and k-nearest neighbors (k-NNs) have been widely used for various classification tasks due to their simplicity and effectiveness [33]. ResNet, Inception, and VGG are notable CNN architectures known for their high accuracy and robustness in image classification tasks [34]. Despite their strengths, these models can face challenges when dealing with class imbalances and subtle differences between categories.

To address these challenges, we further utilized the MaskFormer model for image classification as well. MaskFormer is a state-of-the-art approach that integrates both segmentation and classification into a unified framework, leveraging the power of transformers for comprehensive image analysis. This model is particularly adept at handling the complexities of medical imaging, where precise lesion boundary delineation and accurate classification are critical. More specifically, MaskFormer’s architecture is based on transformers, which are renowned for their ability to capture long-range dependencies and contextual information within an image. Unlike traditional convolutional neural networks (CNNs), which may struggle with the intricate spatial relationships necessary for accurate segmentation, transformers excel at understanding the broader context and finer details of an image. This capability is crucial for differentiating between subtle variations in lesion characteristics and for segmenting lesions pixel by pixel with high precision.

Additionally, MaskFormer’s unique mask-classification framework allows it to simultaneously segment and classify objects. It predicts a set of binary masks, each associated with a class label, enabling it to segment the image into precise shapes of lesions while also determining their classification (normal, benign, or malignant). This dual functionality ensures that the lesion boundaries are accurately defined and classified, enhancing the model’s ability to distinguish between different lesion types and their respective clinical significance.

By leveraging MaskFormer, our study benefits from its advanced architecture and unified approach to segmentation and classification. MaskFormer’s adaptability and precision make it an ideal choice for meeting our classification objectives and advancing the diagnostic capabilities in breast lesion assessment, as lesion shapes and internal characteristics can vary widely.

### 3.4. Beast Lesion Detection Architecture

**Figure 1:**
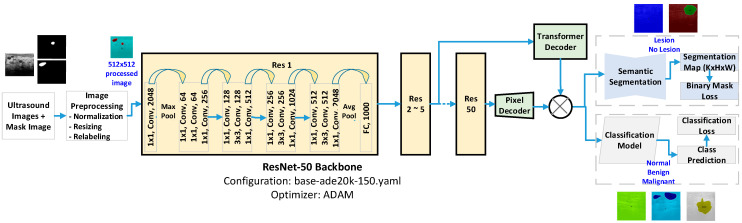
Architectural diagram of MaskFormer-based breast lesion detection and classification of ultrasound images.

The overall architecture of MaskFormer, as illustrated in Figure 1, integrates various components to achieve the precise and accurate identification of lesions for segmentation and classification. Firstly, the input to our model comprises breast ultrasound images along with corresponding mask images indicating the regions of interest (ROIs), lesions. Before the images are fed into the model, they undergo a series of preprocessing steps, including normalization, resizing, and relabeling. This ensures uniformity in the input data and prepares them for effective processing in the subsequent layers.

At the core of our architecture lies the ResNet-50 backbone, configured with the base-ade20k-150.yaml settings and optimized using the ADAM optimizer, as shown in Figure 1. ResNet-50 is a deep convolutional neural network (CNN) known for its robustness in extracting high-level features from input images. It consists of several layers, including convolutional, batch normalization, and activation layers, organized into residual blocks. The ultrasound images, after preprocessing, are fed into the ResNet-50 backbone. The network processes the images through multiple stages, extracting hierarchical features that capture both low-level details and high-level semantic information.

Following the ResNet-50 backbone, the extracted features are passed to a transformer decoder. The transformer architecture is renowned for its capability to capture long-range dependencies and contextual relationships within the image data. Concurrently, a pixel decoder processes the features to refine the segmentation map at a pixel level. This decoder helps in achieving the precise delineation of lesion boundaries by reconstructing the segmented output from the high-level features.

The refined features from the pixel decoder are utilized by the segmentation head to generate a segmentation map. This map indicates the presence or absence of lesions in each pixel of the ultrasound image, as shown in Figure 1. To ensure accurate segmentation, a binary mask loss function is employed. This loss function penalizes discrepancies between the predicted segmentation map and the ground truth mask, thereby guiding the model to improve its segmentation accuracy.

Parallel to the segmentation process, the high-level features extracted via the ResNet-50 backbone are also fed into a classification model. This model is responsible for categorizing the lesions into three classes: normal, benign, and malignant, as illustrated in Figure 1. The classification head outputs the class predictions, which are compared against the ground truth labels using a classification loss function. This function helps the model differentiate effectively between normal, benign, and malignant lesions, enhancing its diagnostic reliability.

The final outputs of the model include a segmentation map that highlights the lesion regions and a class prediction indicating the nature of the lesion. Overall, our MaskFormer-based architecture leverages the strengths of ResNet-50 and transformers to achieve superior performance in the segmentation and classification of breast lesions. The integration of semantic segmentation and classification within a unified framework allows for precise lesion boundary delineation and accurate classification, making our approach highly suitable for aiding in the early detection and diagnosis of breast cancer.

### 3.5. Evaluation Parameters

In assessing the performance of our segmentation and classification models, several key metrics are utilized to quantify their accuracy and effectiveness. These metrics play a crucial role in evaluating the reliability and precision of our results in identifying and categorizing breast lesions. Equations (1)–(5) are the statistical representations to calculate the mean average precision (mAP), mean average recall (mAR), mean average specificity (mASpecificity), mean average sensitivity (mASensitivity), and mean F1 score (mF1), respectively, for our proposed models.

Mean average precision (mAP) is a metric that aggregates the precision across different categories (normal, benign, and malignant). It is calculated as the average of the precisions for each category, quantifying the model’s ability to correctly classify each type of lesion when it predicts them as such (*TP_n_*, *TP_b_*, and *TP_m_*) as detailed in (1). A high mAP indicates that the model maintains high precision across all categories, which is crucial for ensuring that identified lesions are pathologically significant.

(1) $$mAP=\frac{1}{3}\left(\frac{TP_{n}}{TP_{n}+FP_{n}}+\frac{TP_{b}}{TP_{b}+FP_{b}}+\frac{TP_{m}}{TP_{m}+FP_{m}}\right)$$

(2) $$mAR=\frac{1}{3}\left(\frac{TP_{n}}{TP_{n}+FN_{n}}+\frac{TP_{b}}{TP_{b}+FN_{b}}+\frac{TP_{m}}{TP_{m}+FN_{m}}\right)$$

(3) $$mASpecificity=\frac{1}{3}\left(\frac{TN_{n}}{TN_{n}+FP_{n}}+\frac{TN_{b}}{TN_{b}+FP_{b}}+\frac{TN_{m}}{TN_{m}+FP_{m}}\right)$$

(4) $$mASensitivity=\frac{1}{3}\left(\frac{TP_{n}}{TP_{n}+TN_{n}}+\frac{TP_{b}}{TP_{b}+TN_{b}}+\frac{TP_{m}}{TP_{m}+TN_{m}}\right)$$

(5) $$mF1=\frac{1}{3}\left(\frac{2\times P_{n}\times R_{n}}{P_{n}+R_{n}}+\frac{2\times P_{b}\times R_{b}}{P_{b}+R_{b}}+\frac{2\times P_{m}\times R_{m}}{P_{m}+R_{m}}\right)$$

Here,$\;TP_{n},\;TP_{b}\;,\;\mathrm{and}\;TP_{m}$ denote true positive for normal, benign, and malignant images.$\;TN_{n},\;TN_{b}\;,\;\mathrm{and}\;TN_{m}$ denote true negative for normal, benign, and malignant images.$\;FP_{n},\;FP_{b}\;,\;\mathrm{and}\;FP_{m}$ denote false positive for normal, benign, and malignant images.$\;FN_{n},\;FN_{b}\;,\;\mathrm{and}\;FN_{m}$ denote false negative for normal, benign, and malignant images.$\;P_{n},\;P_{b}\;,\;\mathrm{and}\;P_{m}$ denote precision for normal, benign, and malignant images. $R_{n},\;R_{b}\;,\;\mathrm{and}\;R_{m}$ denote recall for normal, benign, and malignant images.

Mean average recall (mAR) measures the average recall across all categories. It signifies the model’s ability to detect each type of lesion among all actual cases of that type, thus minimizing false negatives, as shown in (2). A high mAR ensures that the model consistently identifies lesions across all categories.

Equation (3) measures the mean average specificity (mASpecificity) as the average specificity across all categories. It quantifies the model’s ability to correctly identify normal cases as negative and distinguish them from benign and malignant cases. High mASpecificity ensures that the model consistently avoids false positives across all categories.

mASensitivity measures the average sensitivity across all categories via Equation (4). It signifies the model’s ability to correctly detect each type of lesion among all actual cases of that type. High mASensitivity ensures that the model consistently identifies true positives across all categories, minimizing the risk of missing significant lesions.

The mean F1 score (mF1) is the harmonic mean of precision and sensitivity, offering a balance between the two metrics. It provides a single metric to gauge the overall performance of the model in classifying lesions accurately, considering both precision and recall simultaneously, as detailed in Equation (5). A high mF1 ensures that the model maintains a good balance between precision and recall across all categories, indicating robust performance in detecting and classifying lesions.

Each of these metrics plays a critical role in evaluating the segmentation and classification of breast lesions in ultrasound images. They collectively provide a comprehensive assessment of model performance, aiding in the refinement and validation of our methodology.

### 3.6. Software Environment

For the development and training of our MaskFormer model focused on breast lesion segmentation and classification, we utilized Python 3.12.4 as the primary programming language. The core framework employed was MaskFormer, chosen for its advanced transformer-based architecture tailored to semantic segmentation tasks. The supporting libraries included the following: Detectron2 0.6, an open-source object detection library developed by Facebook AI Research, used for various computer vision tasks; NumPy 1.26.3, for efficient numerical computations; OpenCV-Python 4.10.0.84, for image processing tasks; and scikit-learn 1.5.1, for evaluating model metrics.

Our virtual environment was run on a Windows 11 operating system, which was optimized for compatibility with our hardware configuration. Despite the hardware setup being less robust, it successfully facilitated the training and execution of MaskFormer. The hardware included an Intel Core i7-10700K CPU with 8 cores and a maximum clock speed of 3.80 GHz, 8 GB of Samsung DDR4 RAM, an NVIDIA GeForce GTX 1060 3 GB GPU, and storage with 55.61 GB free on the C: drive and 976.36 GB free on the D: drive, totaling a 2 TB NVMe SSD. This setup, while not the most advanced, enabled us to effectively run and train MaskFormer, demonstrating its capability to deliver high performance even with limited resources, albeit with longer training times.

## 4. Results and Discussions

In the initial stages of our research, we explored the U-Net architecture for breast lesion segmentation in ultrasound images, evaluating its performance using the binary cross-entropy loss and accuracy metrics. We observed that the loss reduction was primarily limited to the first epoch, with subsequent epochs showing minimal improvement, indicating that U-Net struggled to effectively segment the lesions. To enhance performance, we integrated an additional convolutional neural network (CNN) for classification tasks, aiming to classify the segmented regions into normal, benign, and malignant categories. Despite this combined approach, the model did not achieve the desired improvement, as the loss plateaued over multiple epochs. Realizing these limitations, we transitioned to using the MaskFormer architecture, which leverages transformers known for capturing long-range dependencies and contextual relationships. MaskFormer supports both segmentation and classification within a unified framework, and upon training and testing, it exhibited a more consistent and substantial decrease in loss over epochs. This indicated a more effective learning process and better adaptation to the data. MaskFormer demonstrated superior performance in both segmentation accuracy and classification reliability compared to the U-Net and CNN approaches, leading us to continue to improve our research with this architecture due to its robust capabilities in handling breast lesion detection and diagnosis.

### 4.1. Image Preprocessing

In the image preprocessing stage, our goal was to standardize and prepare the ultrasound images and their corresponding masks for effective model training. Initially, we performed normalization, which involved scaling pixel values to a common range in order to enhance the model’s learning efficiency. Each ultrasound image and its mask were resized to a uniform dimension of 512 × 512 pixels in order to ensure consistency across the dataset. The images were then converted to grayscale in order to reduce computational complexity while preserving essential features for analysis.

**Figure 2:**
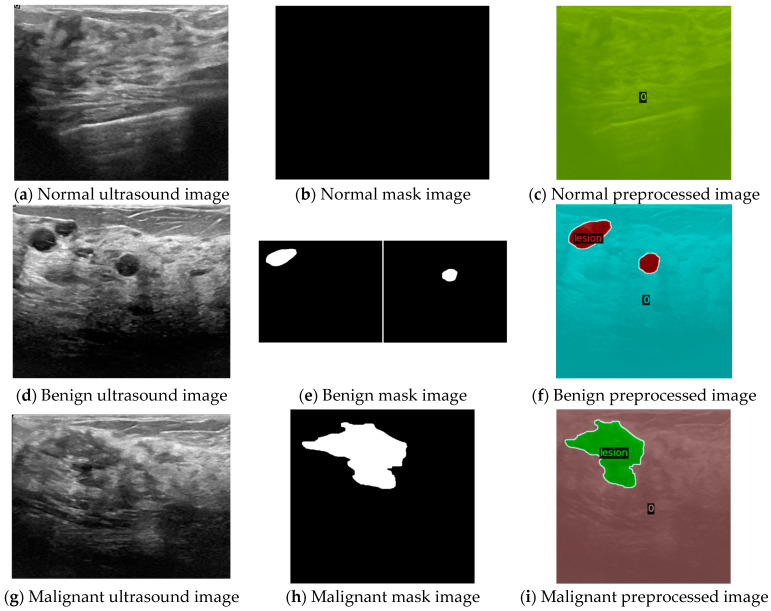
Preprocessing of ultrasound and mask images to produce processed images of 512 × 512 size with labels after normalization: (**a**–**c**) normal ultrasound image, mask image, and preprocessed image; (**d**–**f**) benign ultrasound image, mask image, and preprocessed image; (**g**–**i**) malignant ultrasound image, mask image, and preprocessed image.

For segmentation tasks, the masks were prepared with two classes: ‘no lesion’ (0) and ‘lesion’ (1). This binary classification allowed the model to differentiate between the presence and absence of lesions in ultrasound images. For example, in Figure 2a,b, a normal ultrasound image with no lesions corresponds to an entirely black mask, indicating no regions of interest.

When handling images with multiple lesions, like those shown in Figure 2d,e, we combined multiple mask images into a single mask to indicate all present lesions for the final preprocessed mask 2f. This combination was achieved through operations such as XOR, ensuring that each pixel in the combined mask correctly represented the presence of lesions.

To ensure that our dataset was suitable for training, testing, and validation, we split the data into a ratio of 8:1:1, resulting in 79 images each for testing and validation. This split ensured that our models were trained on diverse and representative samples, allowing for a robust evaluation of their performance. Overall, this preprocessing approach provided a solid foundation for our subsequent experiments with MaskFormer.

### 4.2. Image Segmentation Results

For our image segmentation using the MaskFormer model, we iterated over various hyperparameters to identify the optimal configuration for our segmentation tasks. We continued using the ADAM solver throughout our training, as it had previously proven to be the most effective. Initially, we set the learning rate to 0.001, as indicated in Table 3, which provided a strong baseline performance. However, to potentially refine the model’s adjustments and improve accuracy, we experimented with reducing the learning rate. We decreased it to 0.00025, hypothesizing that a lower learning rate might allow for more precise updates to the model parameters. Despite this, this change resulted in decreased performance, as reflected by a drop in mean average recall (mAR) from 0.807 to 0.621. Table 3 presents these quantitative results from our experiments, showcasing how each configuration impacted the model’s performance across various metrics, such as mAP, mAR, mASpecificity, mASensitivity, and mF1 Score.

Realizing that our GPU could handle a larger batch size, we increased the number of images per batch from two to four. Concurrently, we further reduced the learning rate to 0.0001 and increased the number of iterations to 160,000, assuming these changes might help the model better capture the complexity of the segmentation task. This combination yielded the best results, achieving an mAR of 0.838 and an mF1 of 0.912, which indicate a balanced performance.

**Figure 3:**
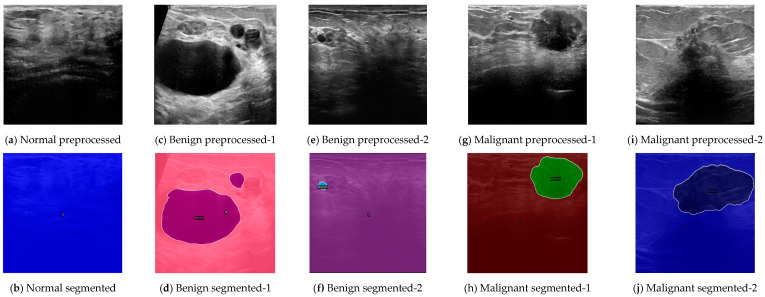
MaskFormer-based segmentation output images for (**b**) normal case, (**d**) benign case with two lesions, (**f**) benign case with very small lesion, (**h**) malignant case, and (**j**) very unclear malignant case.

Figure 3 demonstrates the qualitative results of our strongest segmentation model, effectively segmenting lesions across various cases, including normal, benign, and malignant instances. For the normal case, as shown in Figure 3a, the preprocessed image initially appears to depict potential lesions to the visual eye. However, the AI model correctly segmented it as lesion-free, as indicated in Figure 3b. This shows the model’s accuracy in distinguishing true lesions from normal tissue.

In the benign case with multiple lesions, depicted in Figure 3c, the ultrasound image reveals two distinct lesions. The AI model accurately segmented these lesions into two separate regions, as seen in Figure 3d. This illustrates the model’s capability to handle cases with multiple lesions effectively. When analyzing a benign case with a small lesion, represented in Figure 3e, the lesion is visually difficult to detect due to its size. Nevertheless, the model precisely identified and segmented this small lesion, as shown in Figure 3f, showcasing the model’s sensitivity to detect even minute abnormalities.

Figure 3g presents a malignant case with low contrast conditions. Despite the low contrast, which complicates visual detection, the AI model successfully identified the malignant lesion, as shown in Figure 3h, demonstrating its robustness and reliability under less-than-ideal imaging conditions. Another visually ambiguous malignant case is depicted in Figure 3i, where the ultrasound image presents challenges due to low contrast and image clarity. The AI model accurately segmented the lesion in this instance, as shown in Figure 3j, further demonstrating its robustness.

Overall, through iterative experimentation and the fine-tuning of hyperparameters, these results collectively illustrate MaskFormer’s exceptional ability to accurately segment images and identify lesions, even in challenging conditions, highlighting its potential as a valuable tool in practical medical diagnostics.

### 4.3. Image Classification Results

We continued using the ADAM solver for the classification of breast lesions, leveraging the capabilities of MaskFormer to simultaneously classify and segment lesions pixel by pixel. During training, we iteratively adjusted the hyperparameters to optimize the model’s performance. Initially, we started with a learning rate of 0.001, consistent with the first learning rate used for segmentation, and we set the number of iterations to 160,000, as shown in Table 4. This configuration provided a solid baseline, yielding reasonable results with a mAP of 0.870 and mASpecificity of 0.913, indicating a smaller number of false positives.

However, to enhance performance, we increased the learning rate to 0.0015 while maintaining the batch size. This adjustment aimed to facilitate faster convergence under the assumption that a higher learning rate might help the model perform more significant updates during training. This approach resulted in improved metrics, as reflected in a higher mAP of 0.903, mASpecificity of 0.917, and an mF1 of 0.860, shown in Table 4.

Next, we experimented with a lower learning rate of 0.0002 and increased the images per batch to four, which improved the result slightly. But once we reduced the learning rate to 0.0001 and doubled the number of iterations to 320,000, we observed a significant improvement in the results. This configuration yielded the best results, achieving the highest mAP of 0.943, mASpecificity of 0.937, and an mF1 of 0.893, indicating well-balanced model performance across all categories as summarized in Table 4.

**Figure 4:**
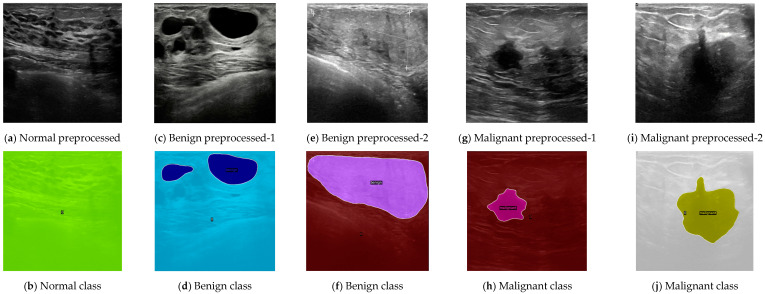
MaskFormer-based classification model output images for (**b**) normal case, (**d**) benign case with two lesions, (**f**) benign case with very large and unclear lesion, (**h**) malignant case, and (**j**) unclear malignant case.

Figure 4 demonstrates the qualitative results of our top-performing classification model. The figures illustrate the model’s ability to accurately differentiate between muscle tissue and lesions and correctly classify various types of lesions under challenging conditions. In Figure 4a, the ultrasound image shows what might visually appear to be multiple lesions. However, the model correctly identified this as normal muscle tissue, as demonstrated in Figure 4b (label 0), indicating the normal case (no lesion). This result showcases the model’s ability to distinguish between normal muscle tissue and breast lesions.

Figure 4c presents an ultrasound image with a benign case involving multiple lesions. The model accurately segmented and classified these lesions as benign, as shown in Figure 4d, again demonstrating the model’s effectiveness in handling cases involving multiple lesions. In Figure 4e, the ultrasound image might look like a normal case with no lesion due to an unclear anatomical image. Despite this, our model successfully detected the very large benign lesion and accurately classified it as benign, as shown in Figure 4f. This result is particularly noteworthy because, while large lesions might often be mistaken for malignant, our model correctly identified them as benign.

It is also worth noting that Figure 4i presents a lesion in an ultrasound image with shadowing. Despite the challenging visual conditions, the AI model accurately segmented the image and classified the lesion as malignant.

**Figure 5:**
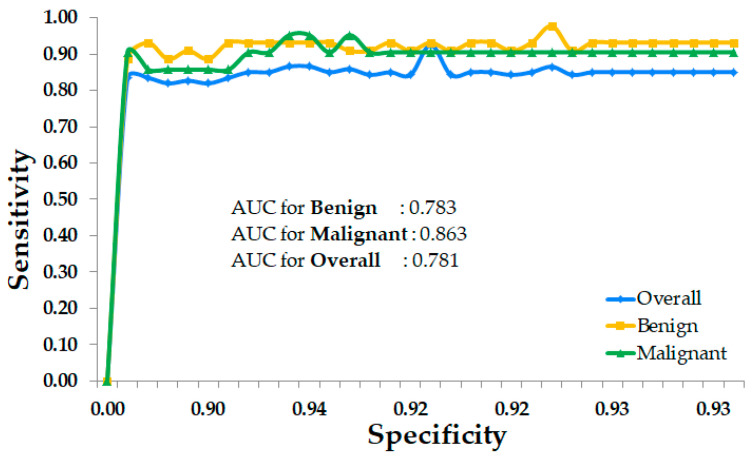
AUC for benign (yellow), malignant (green), and overall (blue) breast lesion detection (segmentation and classification) using MaskFormer model.

As shown in Figure 5, the area under the curve (AUC) values for the best segmentation and classification model using MaskFormer are 0.783 for benign cases, 0.863 for malignant cases, and 0.781 for the overall model. The AUC value for malignant cases indicates a high ability of the model to correctly distinguish malignant lesions from benign and normal breast lesions, which is crucial to notice in the early stages. The benign and overall model AUC also reflect strong performance, demonstrating the model’s ability to classify breast lesions accurately. These high AUC values across categories indicate that the model effectively balances sensitivity and specificity, minimizing both false positives and false negatives. Overall, these examples illustrate how our model performed exceptionally well, even under challenging conditions. The ability to accurately identify and classify lesions can greatly assist in medical diagnoses, providing valuable support to healthcare professionals in their decision-making processes.

## 5. Discussion

In our study, we evaluated the performance of the MaskFormer model for the detection and classification of breast lesions, comparing it with previous AI models used for segmentation and classification tasks. The results demonstrate that MaskFormer is superior in several key metrics, as shown in Table 5, establishing it as the most effective model for this critical diagnostic application.

MaskFormer achieved a benign precision of 0.880, which, while slightly lower than the 0.898 achieved using the YOLO V3-anchor model [25], still indicates strong performance. The lower benign precision suggests that MaskFormer identified more benign cases that were actually normal or malignant, likely due to its high sensitivity in detecting potential lesions and erring on the side of caution. Importantly, MaskFormer achieved the highest benign recall of 0.980 compared to the anchor-free network, YOLO V3-anchor, and YOLO V3-res [24,25]. This high recall is crucial in medical diagnostics, as it ensures that benign lesions are rarely missed, providing confidence that almost all benign cases are correctly identified and reducing the risk of undetected benign lesions.

In terms of malignant lesion detection, MaskFormer excelled with a malignant precision of 0.950. This high precision indicates that the model is highly accurate in identifying malignant lesions, minimizing the occurrence of false positives, when benign or normal cases are mistakenly classified as malignant. Additionally, MaskFormer’s malignant recall stood at 0.900, showcasing its reliability to correctly identify actual malignant cases. This high recall is vital for ensuring that most malignant cases are detected early, which is essential for effective treatment and better patient outcomes.

The mean average precision (mAP) of MaskFormer was 0.943, the highest among all models compared. This high mAP demonstrates MaskFormer’s balanced performance across both benign and malignant lesion detection, effectively combining high precision and recall. Such a balanced and high overall performance is indicative of MaskFormer’s reliability in breast lesion detection and classification compared to other models [24,25].

Additionally, when comparing the performance of MaskFormer with models that used different datasets, it becomes evident that MaskFormer excels in both malignant precision and recall. For example, the SSD300+ZFNet model [22] achieved an accuracy rate (AR) for malignant detection with an average precision rate (APR) of 97.56% but a recall of only 58.96%. The lower recall rate shows that many of the lesions that are actually malignant were missed, which could be detrimental to early detection. In contrast, MaskFormer’s higher recall ensures that malignant lesions do not go undetected. Furthermore, YOLOv3 [23] achieved a recall of 0.835 and a precision of 0.759, which fall short of MaskFormer’s balanced performance, with a malignant precision of 0.950 and a recall of 0.900.

The results unequivocally demonstrate that MaskFormer outperforms the other models in key performance metrics. If we had removed the four outlier images from our normal dataset, we could have achieved a 100% detection rate in normal cases using our best models within MaskFormer. With the highest benign recall and malignant precision, along with the highest overall mAP, MaskFormer stands out as the most reliable and effective model for breast lesion detection and classification in our study. These findings solidify MaskFormer’s position as the best model for this critical diagnostic task, offering balanced and highly accurate performance that is essential for clinical applications.

## 6. Conclusions

Our study underscores the significant advancements in breast lesion detection and classification achieved with the MaskFormer model for ultrasound images. Supporting physicians, especially new practitioners, MaskFormer can identify lesions of varying shapes and sizes, including multiple lesions that are easily missed by the human eye. Unlike humans, who cannot examine images at the pixel level, AI models excel in this detailed analysis. Initial approaches were limiting, as evidenced by early plateauing in loss and suboptimal learning. In contrast, MaskFormer exhibited continuous improvement, delivering precise segmentation and reducing both false positives and false negatives, as evidenced by its superior recall for benign (0.980) and malignant (0.900) cases, along with enhanced mAP (0.943) values. Breast lesion detection using ultrasound heavily depends on operator skill. MaskFormer addresses this issue associated with ultrasound imaging by providing consistent, operator-independent automatic analysis. Overall, the clinical implications are substantial, as accurate early detection and classification facilitated via MaskFormer in ultrasound imaging can lead to timely and appropriate treatment, enhancing the efficacy of breast cancer screening programs.

## Figures and Tables

**Table 1:** BI-RADS guidelines.

BI-RADS Category	Assessment	Description
Category 0	Incomplete	Need additional imaging evaluation and/or prior examination for comparison
Category 1	Negative	No evidence of cancer
Category 2	Benign	Non-cancerous findings such as cysts
Category 3	Probable benign	Low probability of malignancy (<2%), but follow-up imaging recommended
Category 4	Suspicious	Findings that do not look definitively benign and require biopsy
Category 5	Highly suggestive of malignancy	High probability (>95%) of cancer, requiring immediate biopsy
Category 6	Known, biopsy-proven malignancy	Cancer confirmed via biopsy, necessitating appropriate action

**Table 2:** Commercial AI solutions for breast ultrasound.

Manufacturer	Feature	Description	Reported Accuracy
GE Healthcare [16]	Invenia ABUS	AI-powered BI-RADS classification, user-friendly, seamless-integration GE ultrasound system	Sensitivity: ~93% Specificity: ~80%
Siemens Healthineers [17]	AI-Rad Companion Breast	Automated analysis, integration with imaging workflows, BI-RADS support	Sensitivity: ~87% Specificity: ~83%
Philips [18]	AI Breast Ultrasound Solution	Advanced AI algorithms, integration with Philips ultrasound systems, BI-RADS support	Sensitivity: ~88% Specificity: ~83%
Hologic [19]	Brevera Breast Biopsy System	Real-time imaging and biopsy, AI algorithms for classification	Sensitivity: ~85% Specificity: ~80%

**Table 3:** Segmentation results.

Model	Learning Rate	Images per Batch	Solver	Iterations	mAP	mAR	mASpecificity	mASensitivity	mF1
Segmentation: MaskFormer	0.001	2	ADAM	39,000	1.000	0.807	1.000	0.807	0.893
	0.00025	2	ADAM	39,000	1.000	0.621	1.000	0.621	0.766
	0.0001	4	ADAM	160,000	1.000	0.838	1.000	0.838	0.912

**Table 4:** Segmentation and classification results.

Model	Learning Rate	Images per Batch	Solver	Iterations	mAP	mAR	mASpecificity	mASensitivity	mF1
Segmentation: MaskFormer Classification: MaskFormer	0.001	2	ADAM	160,000	0.870	0.833	0.913	0.833	0.847
	0.0015	2	ADAM	480,000	0.903	0.833	0.917	0.833	0.860
	0.0002	4	ADAM	160,000	0.910	0.847	0.927	0.847	0.870
	0.0001	4	ADAM	320,000	0.943	0.863	0.937	0.863	0.893

**Table 5:** Result comparisons.

Method	Benign Precision	Benign Recall	Malignant Precision	Malignant Recall	mAP
MaskFormer (proposed)	0.880	0.980	0.950	0.900	0.943
Anchor-free Network [24]	0.816	0.932	0.789	0.889	0.802
YOLO V3-anchor [25]	0.898	0.954	0.639	0.833	0.769
YOLO V3-res [25]	0.851	0.886	0.637	0.889	0.745

## Data Availability

The data are publicly available in the Breast Ultrasound Image Dataset (BUSI).
